# Management of Molar Incisor Hypomineralisation (MIH): A 1-Year Retrospective Study in a Specialist Secondary Care Centre in the UK

**DOI:** 10.3390/children7120252

**Published:** 2020-11-24

**Authors:** Judith Humphreys, Sondos Albadri

**Affiliations:** School of Dentistry, University of Liverpool, Liverpool L3 5PS, UK; sondos.albadri@liverpool.ac.uk

**Keywords:** molar incisor hypomineralisation, paediatric dentistry, poor prognosis first permanent molars

## Abstract

(1) Background: Molar incisor hypomineralisation (MIH) is an enamel defect that affects an estimated 14.2% of children worldwide. Care takes place in primary and secondary care facilities. (2) Aim: To investigate how children with MIH are managed within a specialist centre in the north of England. (3) Method: A retrospective service evaluation within the paediatric dentistry department was registered with the clinical governance unit. Children who attended consultant-led new-patient clinics between 1 January and 31 December 2015 with a diagnosis of MIH were included. The data collected concerned the pre-referral treatment, the history and diagnoses and the treatments completed. (4) Results: Out of 397 records reviewed, 48 (12.1%) had MIH, where 81.3% and 18.8% of patients had severe and mild MIH, respectively. The majority of patients (*n* = 44 (91.7%)) were referred appropriately. Treatment was completed at the specialist centre for 44 (91.7%) patients. Twenty-five (52.1%) patients had an extraction of one or more first permanent molar teeth. Sixteen patients had the extractions at between 8 and 10 years old and 2 had the extractions later as part of an orthodontic plan. (5) Conclusion: Most children had severe MIH and were referred at an appropriate time to facilitate the consideration of loss of poor prognosis of first permanent molars (FPMs). Most children required specialist management of their MIH.

## 1. Introduction

Molar incisor hypomineralisation (MIH) is a developmental defect of enamel causing cream, yellow or brown opacities on the first permanent molars (FPMs) and sometimes also the incisor teeth [1]. It is a common defect, with the worldwide prevalence estimated to be approximately 14.2% [2], and a local prevalence of 15.9% in the northeast of England [3]. The presentation can vary, with some children presenting with mildly affected asymptomatic teeth and others presenting with severely affected teeth that can be painful or carious, in addition to having aesthetic concerns regarding the incisors [4]. Treatment options are varied and sometimes complicated by the young age of the presenting patients, who may struggle to cooperate with restorative and surgical options.

Treatment planning for MIH is dependent on multiple factors, including severity. Prevention should follow national guidelines for children at high risk of caries for both mild and severe cases [5,6], in addition to using desensitising treatments, such as casein phosphopeptide–amorphous calcium phosphate (CPP–ACP) [7,8,9] and silver diamine fluoride [10]. In Europe, a shared care approach between generalists and specialists is recommended [11,12], with mild cases being managed by general dental practitioners (GDPs) in primary care and the more complex cases being managed by specialists in paediatric dentistry [13] or in orthodontics, where FPMs are to be removed [14].

Severe MIH includes teeth that show evidence of post-eruptive breakdown (PEB), caries, severe hypersensitivity or pain, and children who have strong aesthetic concerns [11]. FPMs may require restoration with either composite resins or preformed metal crowns [15]. Glass ionomer cement may be suitable as a fissure sealant and a temporary restoration but are not durable in the long term [16]. Extraction of poor prognosis FPMs when the patient is 8–10 years old may help to achieve good contact and space closure with the adjacent teeth on eruption [17]. The “ideal time” may be later if the patient has a malocclusion, and therefore referral at an early stage for a specialist opinion is important. Children with MIH often have aesthetic concerns when incisors are affected [18]. They may see an improvement in appearance with micro-abrasion, bleaching or localised composite restorations/composite veneers [19,20]. Some studies have reported higher levels of dental anxiety in children with MIH and more frequent behaviour management problems in comparison to unaffected children [21], necessitating the use of general anaesthetic or sedation.

There are many discussion papers detailing the possible management options for MIH, but little information is published regarding the actual treatment carried out by dentists when managing this condition. Taylor et al. investigated the management of poor prognosis FPMs by GDPs and specialists in paediatric dentistry in the UK by asking for their treatment plans for children with poor prognosis FPMs in clinical vignettes. They found that GDPs would prefer to restore FPMs in comparison to specialists, who were more likely to extract these teeth [22]. Jalevik et al. followed a group of children with severe MIH over 10 years and compared their treatment outcomes to non-MIH peers [21,23]. They were more anxious, had a higher DMFT (decayed, missing, or filled teeth) score and had had treatment of FPMs 4.2 times as often.

No studies have been published to the authors’ knowledge regarding the management of patients with MIH in a hospital setting within the UK The aim of this retrospective service evaluation was to investigate the characteristics and management of children with MIH that were referred to a specialist department in the north of England.

## 2. Materials and Methods

The service evaluation was registered with the local clinical governance team. Patient records for all children who attended consultant-led new-patient clinics between 1 January 2015 and 31 December 2015 were requested and reviewed. Once received, records were checked for MIH as a diagnosis. A data collection sheet was developed and piloted. Data was recorded only for those children with MIH.

The following data were extracted from patient records:

- Whether the referrer identified MIH in the referral and whether treatment on MIH teeth had been attempted
- Symptoms, patient concerns and anxiety
- Number of FPMs and incisors affected, and the severity
- Other diagnoses
- Treatment completed
- Number of appointments and timespan of the treatment in months
- Age at the first visit and at the extraction of FPM(s) (if applicable).

Descriptive statistics were used to analyse the data. The Mann–Whitney *U* test for non-parametric data was used to compare the relationship between the severity of MIH and number of teeth extracted, the number of appointments to discharge and the length of the treatment plan in months. All analysis was carried out using descriptive statistics in SPSS version 24 (IBM, Armonk, NY: USA). Significance was set at *p* < 0.05.

## 3. Results

Data collection took place from March to July 2019. A total of 426 children attended consultant-led new-patient clinics in 2015. Twenty-nine patient records could not be found, and one patient had only temporary notes with no new patient assessment present. In total, 397 patient records were reviewed and 48 patients (12.1%) were found to have MIH.

### 3.1. Patient Characteristics

The mean age of the patients at the new-patient clinic was nine years, seven months (range: 5 years to 14 years 10 months). Twenty-six patients were male (54.2%). The majority of patients were referred by their GDP (*n* = 41 (85.4%)).

Only referrers of eight patients (17.0%) explicitly stated MIH as a reason for referral. Other reasons included enamel defects, most commonly hypoplasia (*n* = 31 (66.0%)), caries (*n* = 17 (36.2%)) or another reason, e.g., trauma (*n* = 5 (10.6%)). Treatment of MIH prior to referral had been attempted for 27 patients (57.4%). Previous fluoride varnish was documented for 10 patients, four teeth had been fissure sealed, 36 teeth had had a restoration placed (temporary or permanent), and seven teeth had been extracted prior to referral.

### 3.2. Complaints and Number of Teeth Affected

Most children presented with a complaint related to MIH (*n* = 34 (72.3%)). The most frequent complaint was of pain from MIH-affected teeth (*n* = 11 (23.4%)). Mild sensitivity or aesthetic concerns was a complaint from nine patients (19.1%). Severe sensitivity was a complaint for five patients (10.6%) in cases followed by “crumbling” teeth (*n* = 3 (6.4%)) and failing restorations (*n* = 2 (4.3%)). Patient anxiety was recorded for 35 patients. Of these patients, 20 (57.1%) were recorded as having dental anxiety.

Table 1 shows the number of affected molars and incisors. The most common combination was four affected molars and two affected incisors. Sixteen patients (33.3%) only had affected molars, whilst 31 (66%) had both molars and incisors affected.

Most children had an additional diagnosis alongside MIH (*n* = 30 (62.5%)). Caries in other teeth not affected by MIH were present for 21 patients (43.8%). A second dental anomaly was present for 9 children (18.8%).

### 3.3. Severity

Table 2 shows the number of children with mild or severe MIH according to the European Academy of Paediatric Dentistry (EAPD) guidelines for severity [11]. Mild MIH includes children with mild sensitivity only, no caries, no PEB and only mild concern about the appearance of the teeth. Severe MIH includes children with sensitivity when brushing teeth or eating, toothache, caries, PEB and severe distress related to the appearance of their teeth. The Mann–Whitney *U* test was used to assess the relationship between the severity of the MIH and the treatment length in months from the first to the last appointment, the total number of appointments and the number of extracted teeth. There was a significant relationship between the number of appointments and MIH severity (*p* = 0.015) and the number of teeth extracted (*p* = 0.014), but not the length of the plan (in months) (*p* = 0.92).

### 3.4. Treatment

Treatment was carried out within the specialist centre for 44 children (91.7%), with the remaining children discharged with a treatment plan to their GDP (*n* = 4 (8.3%)). Table 3 shows the treatments completed for all FPMs (tooth level). Only nine patients (18.7%) required aesthetic treatment for incisors. Table 4 demonstrates the treatment modality used for each patient, with some children requiring more than one type.

For the 25 patients who had an extraction of one or more FPM, the mean age at the time of the first extraction was 10 years, 3 months (7 years, 5 months to 14 years, 9 months). Sixteen patients had the extractions at the “ideal time” (64%) (ages of eight to 10 years), 2 had extractions early (8%), 7 had extractions late (28%) and 2 had extractions later than the normal “ideal time” as part of an orthodontic plan (8%).

The treatment completed and the presenting severity of the MIH was assessed to categorise the treatments into those appropriate to be carried out in primary care and those requiring more complex care, indicating referral due to a need for additional facilities (sedation/general anaesthetic) and/or specialist paediatric dentistry care [12]. The majority of patients (*n* = 44 (91.7%)) needed treatment that was appropriate for referral according to UK recommendations, with only three (6.3%) suitable for primary care. One patient required general anaesthetic management of caries in the primary dentition but had mild MIH, which could be managed in primary care.

## 4. Discussion

This service evaluation demonstrated the variability of the presentation and management for children with MIH within a secondary care specialist setting. In the UK, both general dental practitioners and specialists in paediatric dentistry are available to provide care for children with MIH. No previous studies have looked exclusively at the management of children with MIH in a hospital setting. Taylor et al. investigated the management of poor prognosis FPMs using clinical vignettes. UK-based GDPs and specialists in paediatric dentistry were asked to provide a treatment plan for children with poor prognosis FPMs. They found that GDPs would prefer to restore FPMs in comparison to specialists, who were more likely to extract these teeth [22]. Jalevik et al. followed a group of children with severe MIH over 10 years and compared their treatment outcomes to controls [21,23]. Those with severe MIH were more anxious, had higher DMFT and had treatment of FPMs 4.2 times as often as the non-MIH controls.

Only 17% of children were referred explicitly for management of MIH, which demonstrates that the identification of MIH could be improved in primary care. It is not clear from this data whether this may have disadvantaged these children. The majority of patients were symptomatic due to MIH, most frequently complaining of pain, aesthetic concerns or mild sensitivity. Just over half of the patients had dental anxiety, which is similar to normal population levels for children in the UK [24]. Jalevik et al. found that children with MIH were more anxious than controls with caries [21]; however, a study of school children in Brazil found that there was no difference between children with MIH and controls [25]. The children in the Brazilian study were mostly pre-treatment and had a range of severities of MIH, whereas the children in Jalevik et al.’s study had already undergone treatment for severe MIH, which may explain the difference.

Most children had severe MIH (81.3%) that was affecting both incisors and molars. The majority had four affected FPM teeth, and zero to three affected incisors. It has previously been reported that the greater the number of affected teeth, the more severely affected the teeth tend to be in terms of hypomineralisation [11,26], which is in keeping with our results. Most children with severe MIH required either aesthetic treatment, or restoration and extraction to manage FPMs with caries, PEB or severe sensitivity.

In Europe, guidelines recommend a shared approach for the care of children with MIH [7,11]. GDPs should be confident enough to complete prevention and simple restorative treatment for MIH patients, but when the condition is more severe, referral would be appropriate [12]. The British Society of Paediatric Dentistry also recommends that the majority of children with milder forms of MIH should be managed in primary care [13]. In the UK, general anaesthetic and sedation occurs almost exclusively in community and hospital settings [12], and would therefore also require referral. The results from this study show that this guidance was being adhered to by most referring GDPs in 2015, with only 6.3% of children requiring treatment that could be completed in primary care. A small number of children (8.3%) were discharged back to their GDP for treatment in this cohort.

The number of FPMs extracted was significantly related to the severity of MIH. Although the extraction of FPMs in children can sometimes be straightforward, patient anxiety may necessitate referral for behaviour management or pharmacological adjuncts. Furthermore, it is recommended that the opinion of a specialist in orthodontics or paediatric dentistry is sought if a delayed approach (to address a class II or III malocclusion), compensating extractions or general anaesthetic are to be used [14]. Taylor et al. found that GDPs favoured restoration over extraction in comparison with specialists in paediatric dentistry [22]. It may be that dentists are more comfortable restoring FPMs than planning extraction of permanent teeth in young children. Our findings would agree with this, with many providers referring for guidance regarding poor prognosis FPMs. A recent opinion paper questioned whether a lack of clear guidance on what constitutes a poor prognosis FPM may have led to overtreatment with extraction within the UK, and discussed alternative interim and long-term restorative options that may be appropriate in selected cases [27].

It is worth noting that 72% of children in this cohort had an extraction of one or more FPMs at the ideal time, indicating that most providers sought an opinion at the correct time. The mean age at extraction was 10 years, 3 months. In contrast, a multicentre study conducted in 2007 found that the mean age of extraction of poor prognosis FPMs was 10 years, 9 months for all centres, and 11 years 6 months in the same centre as this study [28], which is well beyond the ideal time of eight to 10 years. This preceded national guidelines on a planned loss of poor prognosis FPM teeth and indicates that the guidelines may have been helpful in promoting referral at the correct age and understanding of the factors influencing the timing of the removal of poor prognosis FPMs [14].

There are several weaknesses to the methodology of this study. A retrospective service evaluation was selected as the most time-efficient way of collecting data; however, a prospective approach may have reduced the amount of missing data, both from omitted details in the records and also from patient records that could not be located. The benefit of looking at data from 2015 meant this allowed for sufficient time such that all patients had finished their treatment or had been discharged. Another weakness of the methodology is the small sample size and the fact that data was only collected from one centre in the north of England. This limits the extrapolation of findings more generally. Further data collection could be carried out at other dental hospitals to explore the management of MIH more generally across the whole UK, giving more robust results with increased external validity. This service evaluation could also be repeated in this centre, allowing for an assessment of local trends in referral and management. A control group comprising children with caries could also have been used for comparison.

These patients started their treatment five years ago, and although it was necessary to look this far back in order to ensure all children had completed their plans (one child had treatment over four years), the picture regarding referral may have changed significantly since then. Across the north-west of England, demand for community- and hospital-based paediatric dentistry has increased significantly, leading to an increase in referral-to-treatment times. The ability for GDPs to refer at the right time has become more complex, as they try to factor in potential delays the child may experience before being seen and whilst awaiting treatment. Additionally, although there was little demand for aesthetics in 2015, in the Instagram^®^ era, it is possible that more patients are now referred for aesthetic management to improve their quality of life.

We are now entering a new era of dentistry during the coronavirus disease 2019 (COVID-19) pandemic, with routine dentistry severely affected in the UK Capacity is reduced in both primary and secondary care owing to furlough times that are designed to protect both patients and staff from contaminated aerosols [29]. Certainly, there was a shortfall of specialists in paediatric dentistry prior to the pandemic [30], but the pandemic has made access to community and hospital services difficult for all dental patients, including children with MIH [31]. The authors suggest two strategies to manage this growing storm. GDPs need to be supported and facilitated to be able to undertake more treatments of MIH in primary care, including prompt access to specialist opinions without referral where possible, such that children may continue to have FPMs removed at the ideal time. Many children will still require general anaesthetic or sedation, and therefore, more specialists in paediatric dentistry must be trained and more specialist positions created in order to meet this increasing demand.

## 5. Conclusions

Despite most referrers not explicitly diagnosing MIH, children were referred at an appropriate time to facilitate the consideration of loss of poor prognosis FPMs. Aesthetic management of incisors was required much less frequently. Extraction under general anaesthetic was the treatment of choice for severely affected teeth.

Most children had severe MIH, which is recommended to be managed by specialists and consultants in paediatric dentistry. The findings are positive and demonstrate appropriate referral by the majority of GDPs to this centre.

## Figures and Tables

**Table 1:** Number of affected molar and incisor teeth per child.

		Molar Incisor Hypomineralisation (MIH) Incisors								Total
		0	1	2	3	4	5	6	8	
MIH Molars	1	3	0	0	0	0	0	0	1	4
	2	8	2	2	1	0	0	0	0	13
	3	2	1	0	2	0	0	0	0	4
	4	3	6	9	3	1	1	1	1	25
Total		16	9	11	6	1	1	1	2	47

**Table 2:** Severity of MIH (mild or severe) as per the European Academy of Paediatric Dentistry Guidelines.

Severity	Number of Patients	Percentage
Mild MIH	9	18.8
Severe MIH	39	81.3
Total	48	100.0

**Table 3:** Treatment completed on the molar teeth (per tooth).

Treatment	Number of Teeth	Percentage
Fissure sealant	32	23.4
Flowable composite	2	1.5
Composite	15	11.0
Glass ionomer cement	7	5.1
Preformed metal crowns	7	5.1
Extraction	49	35.8
Review and extraction later	25	18.2
Total	137	100.1

**Table 4:** Treatment adjuncts used.

Anaesthetic Technique	Number of Patients	Percentage
Behaviour management/local anaesthetic	18	38.3
Sedation	10	20.8
General anaesthetic	20	42.6
